# Perception of the study situation and mental burden during the COVID-19 pandemic among undergraduate medical students with and without mentoring

**DOI:** 10.3205/zma001365

**Published:** 2020-12-03

**Authors:** Jennifer Guse, Ines Heinen, Jana Kurre, Sonja Mohr, Corinna Bergelt

**Affiliations:** 1 University Medical Center Hamburg-Eppendorf, Center for Psychosocial Medicine, Department of Medical Psychology, Hamburg, Germany; 2 FOM Hochschule für Oekonomie & Management, Berlin, Germany; 3 University Medical Center Hamburg-Eppendorf, Education and Students' Affairs, Hamburg, Germany

**Keywords:** mentoring, medical education, mental health

## Abstract

**Objectives:** The COVID-19 pandemic caused drastic changes in medical education and might affect students’ mental health and perception of study conditions. Mentoring may have mediating effects by enhancing social support. The University Medical Center Hamburg-Eppendorf (UKE) offers a voluntary general mentoring program (g-mentoring) for all interested students and a mentoring program for students with excellent course results and scientific interest (e-mentoring) We aimed to investigate the mental burden and views of their study situation during COVID-19 among students who did or did not participate in one of the formal mentoring programs.

**Method: **We conducted a cross-sectional online survey (May 2020) examining students’ mental burden using the Patient Health Questionnaire (PHQ-4), and assessing their perception of study conditions and digital teaching using self-developed items.

**Results:** Of 1193 invited students, 543 (45.5%) completed the survey. 35% of those participated in the g-mentoring and 7% in the e-mentoring. 59% did not participate in any program. More e-mentees than g-mentees and nonparticipants reported clinically unproblematic levels of anxiety and depression symptoms. The majority of students (55%) was somewhat worried about the impact of the pandemic on their study situation. Regarding digital teaching students did not feel overburdened by the lack of a fixed schedule and structure, e-mentees even less than g-mentees and nonparticipants. Both g-mentees and nonparticipants were significantly more appreciative of the possible repeated use of the digital teaching materials than e-mentees (both groups M=5.7 vs. M=5.4 in e-mentees, p=.045).

**Conclusion:** The results indicate that while students feel substantially burdened by the situation and the majority worries about the impact of the pandemic on their studies, they also seem to cope well with the digital course format. Study motivation during COVID-19 decreased among the majority of students with and without mentoring. These aspects may be important to address by medical schools interested in developing effective interventions to support students during a pandemic and continuous online teaching.

## Zusammenfassung

**Hintergrund: **Die COVID-19-Pandemie führte zu drastischen Veränderungen in der medizinischen Ausbildung und könnte sich auf die psychische Gesundheit von Studierenden und ihre Wahrnehmung der Studiensituation auswirken. Mentoring könnte einen mediierenden Effekt haben indem es die soziale Unterstützung erhöht. Das Universitätsklinikum Hamburg-Eppendorf (UKE) bietet ein freiwilliges Allgemeines Mentoringprogramm (A-Mentoring) für alle interessierten Studierenden an und ein Mentoringprogramm für exzellente Studierende mit herausragenden Studienleistungen und wissenschaftlichem Interesse (E-Mentoring). Ziel dieser Studie ist es, die psychische Belastung und Wahrnehmung der Studiensituation während der COVID-19 Pandemie von Studierenden zu untersuchen, die an einem der formalen Mentoringprogramme teilnahmen oder nicht teilnahmen.

**Methodik: **Es wurde eine online basierte Querschnittsbefragung durchgeführt (Mai 2020), um die psychische Gesundheit mit Hilfe des Patient Health Questionnaire (PHQ-4) zu untersuchen. Die Wahrnehmung der Studiensituation und der digitalen Lehre wurde mit selbst entwickelten Items erhoben.

**Ergebnisse:** Von 1193 eingeladenen Studierenden nahmen 543 (45.5%) an der Befragung teil. 35% nahmen am A-Mentoring teil und 7% am E-Mentoring. 59% nahmen an keinem Mentoringprogramm teil. Mehr E-Mentees als A-Mentees und Nicht-Teilnehmende gaben klinisch unproblematische Werte für Depression und Angst an. Die Mehrheit der Studierenden (55%) war etwas besorgt über die Auswirkungen der Pandemie auf ihre Studiensituation. In Bezug auf die digitale Lehre fühlten sich die Studierenden durch das Fehlen eines festen Zeitplans und einer festen Struktur nicht überfordert, E-Mentees noch weniger als A-Mentees und Nicht-Teilnehmende. Sowohl A-Mentees als auch Nicht-Teilnehmende schätzten die mögliche wiederholte Nutzung der digitalen Lehrmaterialien signifikant mehr als E-Mentees (beide Gruppen M=5.7 vs. M=5.4 bei E-Mentees, p=.045).

**Schlussfolgerung: **Die Ergebnisse deuten darauf hin, dass sich die Studierenden zwar durch die Situation erheblich belastet fühlen und die Mehrheit über die Auswirkungen der Pandemie auf Ihr Studium besorgt ist, dass sie aber auch gut mit dem digitalen Kursformat zurechtzukommen scheinen. Die Studienmotivation während COVID-19 nahm bei der Mehrheit der Studierenden mit und ohne Mentoring ab. Diese Aspekte könnten für Medizinische Fakultäten, die an der Entwicklung von Interventionen zur Unterstützung von Studierenden während einer Pandemie und kontinuierlicher Online-Lehre interessiert sind, von Bedeutung sein.

## Introduction

The coronavirus disease (COVID-19) global pandemic has brought widespread disruption to medical education [1], [2]. In March 2020 it was agreed in Germany that lectures will predominantly be held in a digital format until further notice [3]. The short-term modification of the learning environment to online teaching and uncertainty caused by COVID-19 might affect both faculty and students [4]. Recent studies report high prevalence of mental health symptoms among health care workers exposed to COVID-19 [5], [6]. Even in the absence of COVID-19, mental distress is common among medical students [7], [8], [9], especially in those with low social support [10], [11]. Since structured mentoring programs may help to promote social support [12], the University Medical Center Hamburg-Eppendorf (UKE) offers a general mentoring program (group mentoring with max. 8 mentees/mentor) to all interested medical students (g-mentoring) from study year 2 onwards as well as an excellence mentoring program (e-mentoring; predominantly 1:1 mentoring) for the 10% of students with excellent academic performance [13], [14]. Due to the pandemic, the mentoring program was transitioned into a digital mentoring offer on a voluntary basis. We investigated the mental burden and perception of study conditions among students who did or did not participate in the program.

## Methods

The mentoring program at the UKE was launched in 2009. Participation is voluntary for mentees (students) and mentors (faculty). Objectives of both (g- and e-mentoring) include but are not limited to providing students with a person of trust they can turn to with questions, enabling mentees to build up a personal network and professional and career progression of mentees. In addition, the e-mentoring aims at early involvement of mentees in research. Topics and frequency of meetings are needs oriented. Further details have been previously reported [13]. Mentoring objectives of the digital mentoring program were the same as before, but more frequent meetings depending on the mentees’ needs during lockdown were recommended. However, most mentors faced increased health care demands in light of COVID-19. Thus not all mentors were able to offer digital or any mentoring.

In May 2020, six weeks after the semester has started, all undergraduate medical students at the UKE were invited to a voluntary anonymous course evaluation. In this context, we conducted a cross-sectional online survey and examined students’ level of mental burden (Patient Health Questionnaire (PHQ-4) [15], German version) as well as their views of their study situation and change of study motivation during COVID-19 (self-developed items). The PHQ-4 is a reliable (Cronbachs α=0.81) and valid screening instrument for young adults [16]. Students also evaluated the predominantly asynchronous online course formats (predominantly slides with audio commentary) on Likert scales (self-developed items). 

We used descriptive statistics to characterize the sample. Group comparisons were carried out using chi2-tests for categorical variables and ANOVAs for differences of means. All analyses were carried out using IBM SPSS version 26. The local ethics board of the Center for Psychosocial Medicine at the UKE approved the study (LPEK-0161).

## Results

596 out of 1193 students in study years 2 to 4 participated in the survey. 53 of those had to be excluded due to missing data in age, gender or mentoring participation, resulting in a final sample of 543 students for analyses.

59% did not participate in any mentoring program, while 35% participated in g-mentoring and 7% in e-mentoring. Significantly more e-mentees than g-mentees and nonparticipants were younger than 26 years, and slightly more e-mentees (60%) reported clinically unproblematic levels of anxiety and depression symptoms (see table 1 (Tab. 1)). 44% of the participants in both the g- and the e-program reported that their mentor offered digital mentoring during the pandemic. 22% in the e-program and 26% in the g-program made use of this offer.

As shown in table 2 (Tab. 2) the majority (55%) was somewhat worried about the impact of the pandemic on their study situation. More e-mentees than g-mentees and nonparticipants reported to be unperturbed (“I am as worried or unworried as before”) and less e-mentees reported to be seriously worried. The rate of students reporting a decreased study motivation was highest among e-mentees (60%). However, these group differences did not reach statistical significance. On average, students reported not to be overstrained by the lack of a fixed schedule and structure due the digital teaching conditions, e-mentees even less so than others. All groups were highly appreciative of the possibility to use digital teaching offers independently of time schedule and location. While both g-mentees and nonparticipants were significantly more appreciative of the possible repeated use of the digital materials than e-mentees, the rate of students reporting to often or very often making use of this offer was highest among e-mentees (see table 2 (Tab. 2)).

## Discussion

COVID-19 poses enormous strains on students as well as on faculty. Especially at a university medical center, faculty members are facing multiple demands such as clinical, teaching and research duties. Still almost half of our faculty mentors spontaneously volunteered to offer digital mentoring to their mentees irrespective of the specific program, whereas other medical schools employed peer mentoring for students during COVID-19 [17]. 44% of the students reported mild to severe symptoms of anxiety and depression. Although this is a cross-sectional study, which does not allow causal statements, the findings stress the impact of COVID-19 and the changes in study conditions on students’ well-being. However, our results indicate that while medical students feel burdened by the COVID-19 situation and the majority worries about the impact of the pandemic on their studies, they also seem to cope well with the digital course format.

Excellent students seem to be on the one hand less frequently mentally burdened and worried about their current study situation due to the pandemic and seem to feel confident in the handling of digital course offers while on the other hand they seem to be more frequently frustrated with the situation and report a decrease in their study motivation. Age might be a confounder regarding the increased number of unproblematic levels of anxiety and depression among e-mentees and should be examined in the future. Excellent students might be particularly ambitious and e.g. preoccupied with doctoral research studies concurrent to their medical courses. During lockdown this was often more difficult to pursue and might account for the more frequent frustration with the situation among those students. In this context, Gotian’s suggestion regarding a change of the mentoring approach during COVID-19 might apply: She strongly recommends to lower expectations while dealing with extreme conditions, to focus on the empathic part of the mentoring relationship, and to listen and acknowledge the mentee’s emotions and perception [18]. Training programs for mentors are well-recognized to increase mentoring competency and enhance effectiveness [19], [20], [21], [22]. Considering Gotian’s recommendations regarding a different type of support during the pandemic and our findings of few differences between students with and without mentoring, this highlights the importance of a tailored mentor training in the near future to support mentors in this new setting of digital mentoring.

This study has several limitations: most importantly, the cross-sectional design does not allow causal statements and the conduct at a single-institution limits the representability.

## Conclusion

The results indicate that while students feel substantially burdened by the situation and the majority worries about the impact of the pandemic on their studies, they also seem to cope well with the digital course format. Regardless of whether students participated in mentoring or not, the majority reported a decreased study motivation since the beginning of the pandemic. Thus, study motivation in light of the drastically changed learning environment due to COVID-19 seems to be an important issue and should be addressed by mentors and faculty in general. The results may be useful for medical schools interested in developing interventions to support students during the pandemic.

## Funding

The mentoring program is funded by the Claussen-Simon-Stiftung, Hamburg.

## Acknowledgements

We would like to thank all students who participated in the study and the evaluation team of the faculty who administered the online survey.

## Competing interests

The authors declare that they have no competing interests. 

## Figures and Tables

**Table 1:**
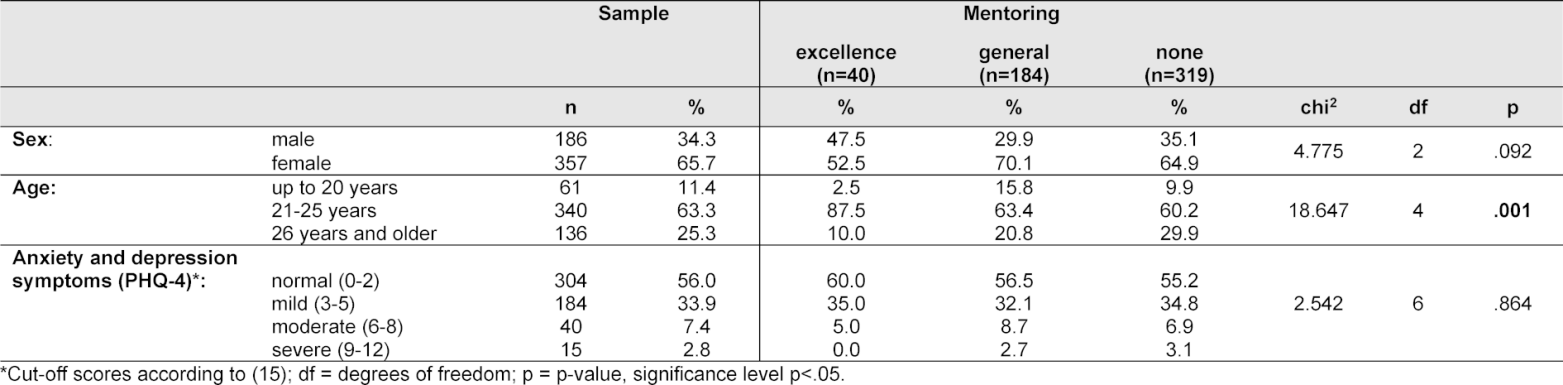
Sample characteristics and anxiety and depression symptoms among n=543 undergraduate medical students

**Table 2:**
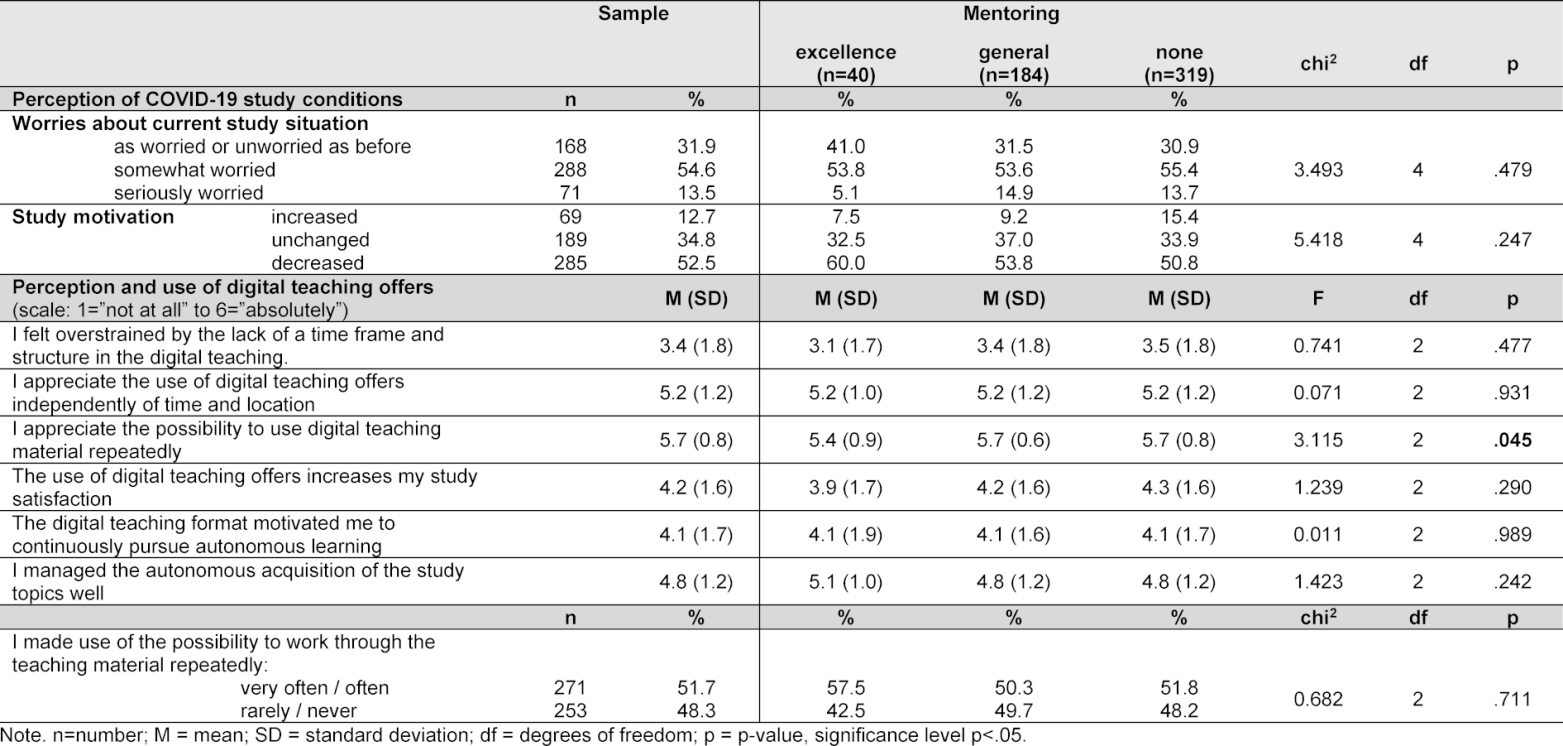
Perception of COVID-19 study conditions and digital teaching offers among n=543 undergraduate medical students
